# Metaphorical Peace: Translating the Women Peace and Security Agenda in the Arab World

**DOI:** 10.1007/s11196-026-10467-0

**Published:** 2026-03-13

**Authors:** Catherine Turner, Sarah Al-Areqi

**Affiliations:** 1 Durham University, Durham, UK; 2 Doctoral Researcher, School of Government and International Affairs, Durham University, Durham, UK

**Keywords:** Peace, International law, Women peace and security, Arab world

## Abstract

Since the end of the Cold War the international peace architecture has grown exponentially. A progressively more detailed and conceptually broad understanding of peace has emerged to underpin peacemaking by the UN and other international actors. This definition has been developed through a series of resolutions and reports that articulate a specific understanding of ‘peace’ in which ‘sustaining peace’ requires the prevention of conflict and addressing its root causes. This paper argues that the expansion of this field has led to the situation where the concept of ‘peace’ has become so conceptually stretched that it operates in its current form as little more than a metaphor for a detailed normative international order which is concealed behind the metaphor. However, following nearly two decades of normative consolidation we are witnessing a global pushback against international norms. This dynamic is particularly evident when it comes to the Women Peace and Security agenda where language has emerged as a key terrain for the political contestation of norms. This paper compares the English and Arabic translation of key WPS resolutions to demonstrate the difficulties of ‘translating’ complex concepts related to peace across linguistic and cultural contexts. In doing this it provides a valuable resource for those concerned with the survival of Women Peace and Security as a global agenda.

## Introduction

Since the end of the Cold War the international peace architecture has grown exponentially. A progressively more detailed and conceptually broad understanding of peace has emerged to underpin peacemaking by the UN and other international actors. This definition has been developed through a series of resolutions and reports that articulate a specific understanding of ‘peace’ in which ‘sustaining peace’ requires the prevention of conflict and addressing its root causes [1]. The growth of this body of texts emerging from the UN system has been accompanied by the emergence of a professionalised field of practice in which expertise is required to properly ‘translate’ the requirements of text to practice. This paper argues that the expansion of this field has led to the situation where the concept of ‘peace’ has become so conceptually stretched that it operates in its current form as little more than a metaphor for a detailed normative international order which is concealed behind the metaphor. This has had consequences for the ability of the concept to travel across diverse geographic and cultural contexts. To demonstrate this linguistic process and its implications in practice we offer a detailed analysis of the ‘translation’ of the Women Peace and Security (WPS) agenda in the Arab World. The WPS agenda was first institutionalised in the year 2000 with the adoption of United Nations Security Council Resolution 1325 (UNSCR 1325). This landmark resolution provided for the first time that women should be included in all endeavours to secure peace and security [2] and laid the textual foundation for the normative development of the agenda through the adoption of 10 subsequent resolutions that set out the requirements of women’s full, equal and meaningful participation [3]. Implementation of the WPS agenda has become a key element of the UN’s approach to ‘inclusive’ peace, which until 2019 continued to grow in reach and influence. However, following nearly two decades of normative consolidation we are now witnessing a global pushback against international norms that has made it difficult to use the language of the WPS agenda in some forums. Language itself has emerged as the primary terrain for political contestation of norms, leading many international organisations, governments and NGOs to revise their policies on language use [4]. UNSCR resolutions are a key battleground in the current contestation of language and norms both at the UN and in national contexts where there has been pushback against the WPS agenda [5]. When it comes to WPS, language matters.

In this paper we analyse the concept of ‘peace’ as a metaphor in the United Nations system. Using the case study of the Women Peace and Security agenda we address two research questions. First, how has the semiotic content of the UNSC resolutions thickened over time to construct a legally relevant normative order of gender equality? Second, what does the translation into Arabic of these commitments tell us about the pragmatic aspects of metaphorical peace? The article proceeds in three sections. First, we outline the gradual evolution of a ‘thick’ concept of peace in UN policy and practice. Second, we set out our legal linguistic approach to analysing the text and the translation of key WPS documents. Third, we compare the English and Arabic translations of the resolutions to demonstrate the semiotic and pragmatic differences between the two. In so doing ewe argue that this comparison of the English and Arabic versions of the official texts and their application in context sheds light on the reasons for resistance to the specific language of the WPS agenda in Arab countries. The aim of the article is *not* to argue that the WPS agenda is incompatible with Arab language, culture and thought, but to demonstrate how the translation of UNSCR resolutions fail to account for the deeper semiotic and pragmatic meaning of key concepts in language.

## The Emergence of ‘Metaphorical Peace’

The starting point for an analysis of the translatability of the language in peacemaking and peacebuilding is the definition of peace itself. As Vernon notes, the terminology of peacebuilding is replete with metaphor [6]. The term ‘building’ implicitly conveys the idea that peace is something that can be constructed, following a plan and using the specific tools at our disposal. The language of ‘levels’ that is commonly used to describe multi-track peace processes and the different spaces in which peacebuilding practice operates presents a similar metaphorical quality, suggesting vertical differentiation (and hierarchy) between these spaces. Beyond the concept of building, the term ‘peace’ itself is rich with possibility as a metaphor. For example, we can think of ‘peace’ as the *silence* that comes when the guns stop. Or as being able to *rest* after living with prolonged and acute insecurity. It evokes the leaving behind of a previous state, the cessation or the ending of the conditions that were preventing the achievement of peace.

There is now a rich literature that seeks to define the meaning of peace [7]. From the concept of ‘positive peace’ that introduced a ‘whole of society’ approach to peace [8], various theories have been developed that seek to give greater conceptual depth to the definition of peace beyond the absence of war. These include attempts to develop theories for how peace can be achieved, including agonistic peace [9] and relational peace [10] which focus on the interactions between people in conflict. There are also more substantively oriented theories that seek to link the idea of peace with a particular form of social (re)-ordering that redresses exclusion and inequality. Examples include theories of feminist peace [11], and decolonial peace [12] which centre traditionally marginalised ontologies and epistemologies in how we think about the structural conditions of peace.

In contrast to this literature which has sought to understand the different possible ontologies of peace [13], at the policy level the approach has been semantic, prioritising the development of a fixed meaning behind the text of key UN documents. This redefinition of peace was made possible by the ending of the ‘adversarial decades of the cold war’ and the opening up of a space for conceptualising peace as more than the absence of military threats at the global level [14]. The concept of peacebuilding emerged from the UN’s Agenda for Peace in 1992. This landmark report highlighted the connected aims of the Charter that included not only security, but also justice, human rights and social peace, highlighting ‘an increasing moral perception, finding expression in international law’ that it is the role of the UN to address the deepest causes of conflict: economic despair, social injustice and political oppression [14. para 15]. The whole of society approach to peace required strengthening national domestic institutions and infrastructures [15].

These normative requirements of peace were further supplemented in 2012 when the idea of a ‘comprehensive’ peace agreement was articulated by the UN to capture the need to address a broad range of social issues including power sharing, constitutional reform, justice, human rights and security sector reform through its mediation and peacebuilding activities [16]. Linking the institutional activities of peace and development, the 2016 ‘Sustaining Peace’ report reflects the broadening of the meaning of peace, highlighting the importance of strengthening the rule of law … and promoting sustained and sustainable economic growth, … national reconciliation and unity, including through inclusive dialogue and mediation, access to justice and transitional justice, accountability, good governance, democracy, accountable institutions, gender equality and respect for, and promotion of human rights and fundamental freedoms’ [1. preamble].

The 2016 report is the high-point of concept formation and the clustering of key activities seen as essential for peace [17]. In subsequent reports the emphasis shifts from concept development to implementation, with peace broadly understood as a goal and a process to build a common vision of society, ensuring that the needs of all segments of the population were taken into account[18].

It is this body of increasingly normative textual provisions that provides the basis for a linguistic analysis of peace in international law and policy. We argue that the term peace has become a metaphor for a normative definition that contains both implicit and explicit assumptions about the causes of conflict that must be responded to, as well as a particular ideological belief in what ‘peace’ should look like that draws a semantic border around the word [19, p. 93] and attempts to convey unequivocal meaning independent from context [19, p. 112]. The language that has been used over successive reports, resolutions and guidance notes has provided specific tools for constructing peace, with the meaning being conveyed being that of a ‘liberal’ peace. A linguistic analysis helps to deconstruct some of the core elements of a policy that has embedded the implementation of normative goals through local engagement, revealing the relationship between this approach and the pushback against these norms that we are now experiencing.

## Legal linguistics- ‘Peace’ as a Thick Signifier

Analysing peace policy through the lens of legal linguistics allows for a more focused analysis of both the semiotics of the term peace, as well as the pragmatics of its use in a global context.

### Legal Linguistics – Peace in International Law

**Fig. 1:**
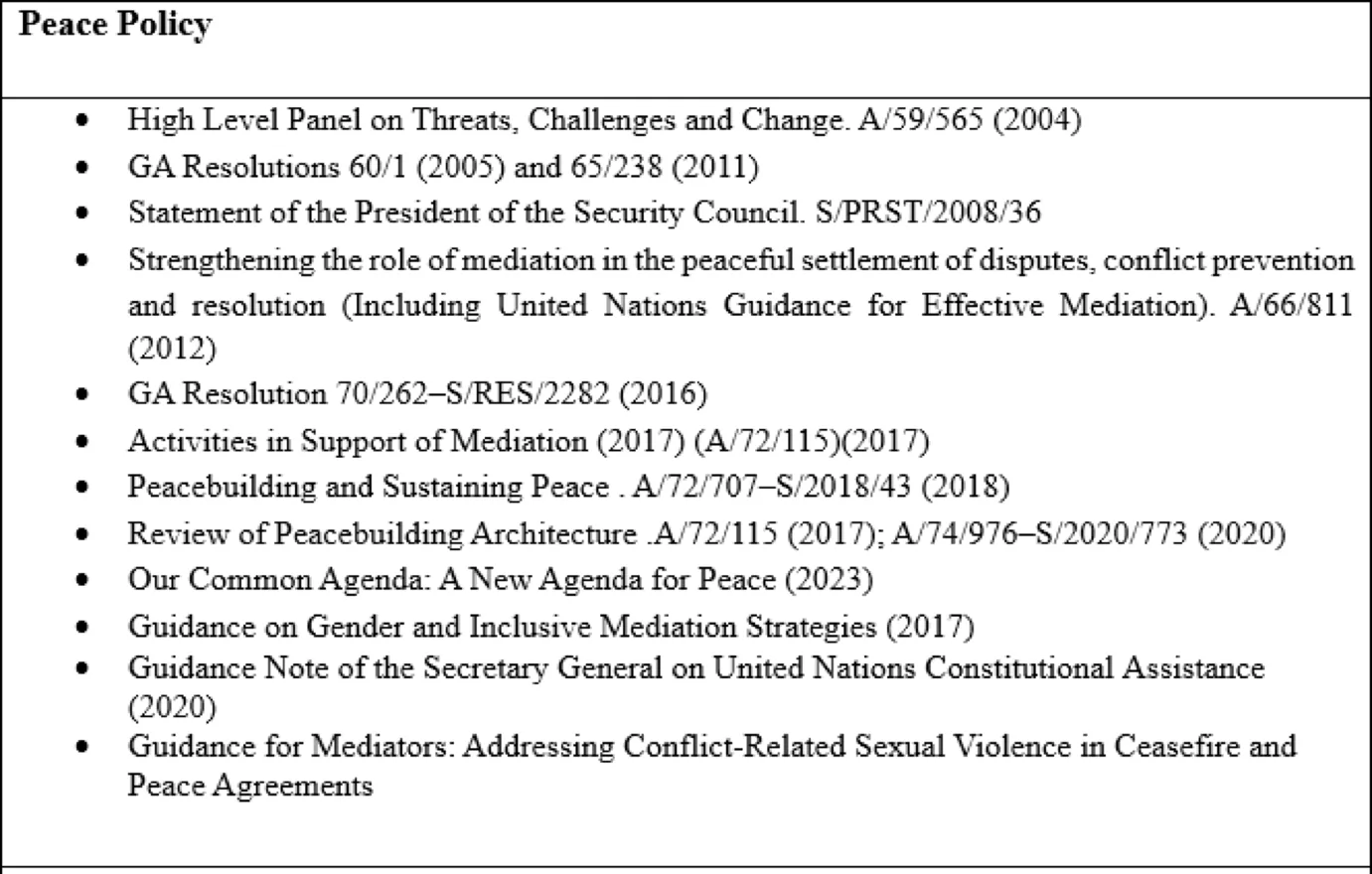
Textual Evolution of Peace Policy

**Fig. 2:**
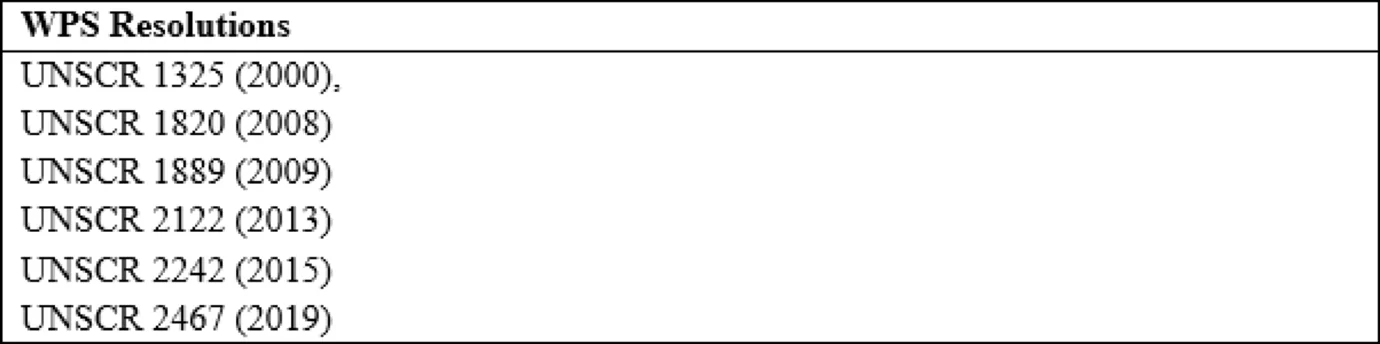
The WPS Resolutions

While the definition and normative requirements of peace in international law are the subject of much broader enquiry, for the purposes of this article we focus on the evolution of the concept of inclusive peace at the UN. This approach allows us to demonstrate the relationship between (i) the text of the UN documents as they have developed over time, (ii) the evolution of a professional field of practice that implements the norms contained in the texts, (iii) and their translation across cultures globally (Figs. 1, 2). 

**Fig. 3:**
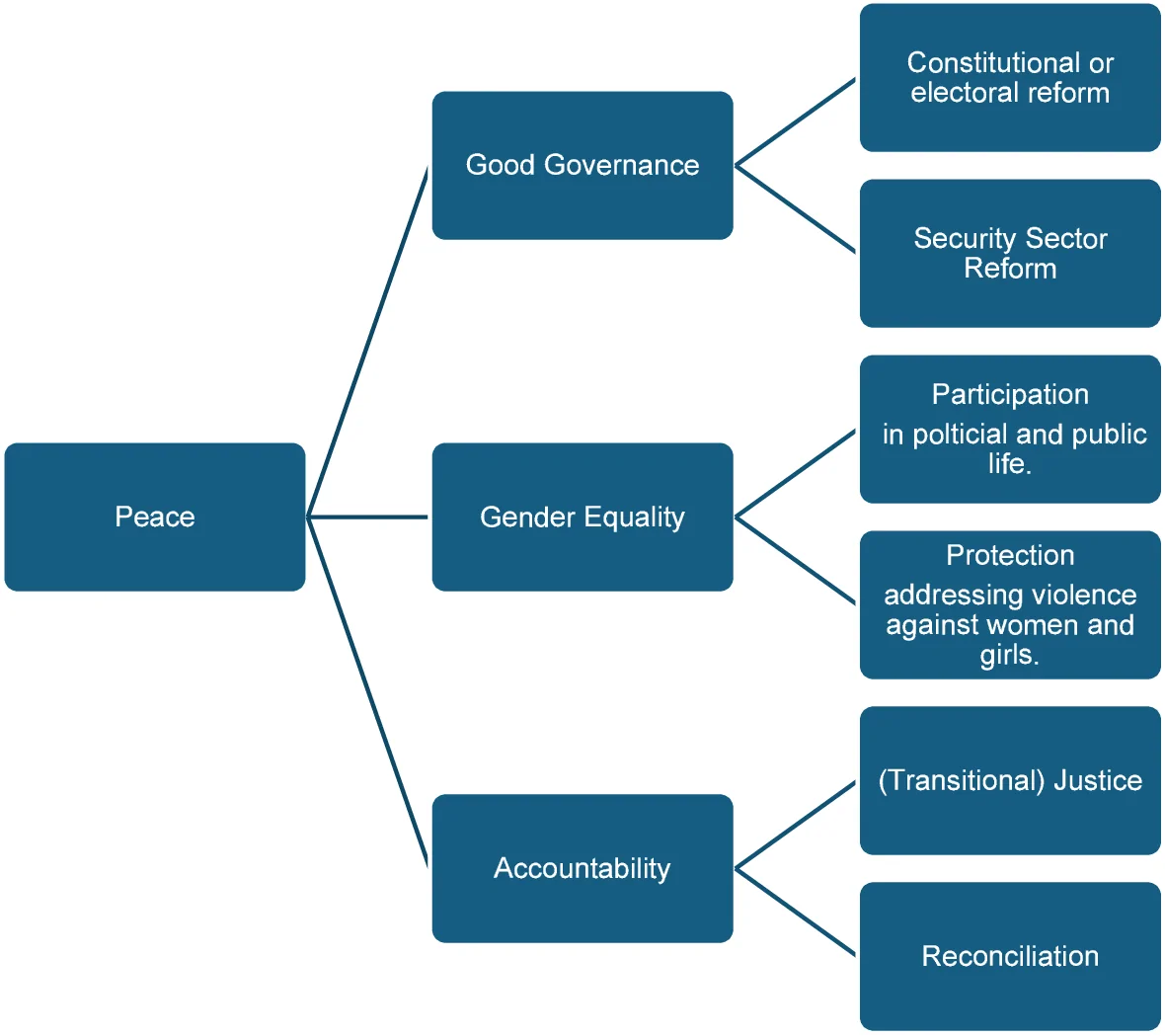
Semiotic Thickening of ‘Peace’

Methodologically we draw on Marcus Galdia’s work on legal linguistics to interrogate the role of international law in constructing the meaning of peace at the UN. Adopting a qualitative legal-linguistic approach we first set out the semantic aspects of peace policy before moving on to consider both a semiotic and pragmatic perspective. This analysis consists of two parts. The first is analysis of the textual corpus that develops the concept of peace at the United Nations. These texts were analysed qualitatively to identify the connections between the term ‘peace’ and the thicker semiotic signifiers that it connoted (Fig. 3).

Our linguistic analysis focuses on texts that create law or normative authority. We understand law in this context to mean (i) a set of prescriptive texts that are (ii) created and implemented in a given context. It should be noted, however, that the texts that we are working with are primarily those of the UN and as such have varying degrees of legal authority. Language plays an important role in the construction of international legal normativity. Official UN documents emerge through institutional processes that seek to reduce highly political or ethical questions to semantic clarity [19, p. 74]. They are the outcome of debate within the relevant institutions, producing a specific communicative effect whereby legal or normative authority is produced through the process of rendering the speech acts of debate into the text of resolutions that are adopted as formal documents of the institution [19, p. 90–91, 20]. From a linguistic perspective, texts such as resolutions of the UN Security Council adopted under Ch VI of the Charter are considered prescriptive- setting out expected actions but falling short of having the full force of law. It is this category into which the WPS resolutions fall.

The second step was to analyse the body of UNSC resolutions that comprise the Women Peace and Security Agenda. Analysing these texts as illustrative examples of the phenomenon of metaphorical peace offers methodological advantages because they are generally regarded as having some normative effect and are often analysed as ‘international law’ [21]. Further, textual analysis of the WPS resolutions is an established methodology in feminist legal studies [20, 22, 3].

The analysis of the resolutions proceeded in three stages. First, qualitative thematic coding identified recurrent signifiers across the English-language resolutions, focusing on participation, protection, and gender mainstreaming. These signifiers were traced diachronically to examine processes of semantic thickening, whereby initially normative formulations acquire institutional, procedural, and juridified density over time. This approach draws on semiotic understandings of policy terms as “thick signifiers” whose meaning is produced relationally within discursive systems [23, 17]. Second, corresponding Arabic provisions were examined comparatively to assess lexical equivalence, semantic shift, and the treatment of polysemy. Particular attention was paid to compact English policy compounds that condense institutional assumptions into technical vocabulary and to how their Arabic renderings redistribute, extend, or compress pragmatic force. This stage engages legal linguistics’ concern with institutional meaning production and the performative character of legal language [19, 24]. Third, policy neologisms were identified and analysed in light of Arabic morphological structure, including triliteral roots and derivational patterns. Terms such as al-nawʿ al-ijtimāʿī (‘gender’) and taʿmīm al-manẓūr al-jinsānī (‘gender mainstreaming’) were examined as constructed lexical imports within global governance discourse.

The study does not employ quantitative corpus linguistics. Instead, it adopts an interpretive and critical approach consistent with feminist analyses of WPS resolutions as negotiated textual artefacts embedded in institutional power relations [20; 22]. English is analysed as the dominant drafting language within UN institutional practice [25], while Arabic is examined as a formally equivalent, yet structurally distinct linguistic system shaped by root-based morphology and relational semantic fields [26, 27]. Arabic terms are presented in transliteration without full diacritics for readability, except where necessary to clarify morphological distinctions. Where analytically relevant, triliteral roots are referenced to illuminate semantic genealogy. The analysis focuses exclusively on official UN Arabic formulations rather than back-translations or field renderings. The objective is not to assess translation accuracy. Rather, it is to examine how the cross-linguistic movement of semantically thick policy signifiers reveals institutional presuppositions, governance expectations, and asymmetries in normative authorship within the global peace architecture.

### Peace as Thick Signifier

Whereas the study of peace policy has been dominated by semantic approaches, adopting a semiotic approach allows us to interrogate the contestations around the signification of ‘peace’ and some of its constitutive elements [17]. In this context the focus is on the institutions of the United Nations, which function as the primary global forum for the promulgation of the norms and standards of peace, justice and human rights. Meaning making in these institutions is not therefore the search for a metaphysical truth that can be rendered semantically intelligible, but the result of discursive contestation [20] where the definition of peace adopted in text depends on its difference from other (excluded) signifiers [23, 28].

Shepherd uses the construct of ‘author-ity’ to demonstrate the dual aspect of both ownership and control of key terms that is produced when meaning is institutionalised in text through these processes [20]. The language breaks free from its context and it used to convey an ‘idealised’ vision of peace to be implemented [5, 29]. This process is exemplified in the growing body of Guidance Notes that have been central to the professionalisation of peacebuilding and mediation. Detailed guidance has been produced on ‘how to’ ensure gender and inclusive mediation strategies which also exist within a broader legal and normative ecosystem. It is not possible to understand the requirements of semantic terms such as ‘accountability’ or ‘gender equality’ without understanding the ways in which these have been formulated and interpreted in other fora. These include the directive texts of ‘hard’ law treaty provisions on the prevention and punishment of genocide, war crimes, or conflict related sexual violence for example, or in the assertive ‘soft’ law General Comments of the Committee on the Elimination of all forms of Discrimination Against Women on women’s rights in armed conflict. While the aim of these texts has been to provide definitional clarity on the normative framework of peacebuilding, the words themselves remain replete with uncertainty, requiring interpretation [30, p. 23–24].

The development of the normative guidance on the requirements of peace has gone hand in hand with the process of ‘professionalisation’ of the field of practice, involving the development of normative guidance, the training of participants in the system, and the cultivation of thematic expertise [31, 5]. While ‘peace’ is the signifier, the work of interpretation is done by the growing class of international professionals who implement the technical requirements of the UN policy framework, often through partnership with local civil society organisations. A ‘linguistically relevant professional knowledge’ imposes rules of the game that mediate social interactions [19, p. 31]. Priority is given to some solutions, actors and interests at the expense of others [28]. This is a process that exemplifies how particular ways of thinking and being are treated as neutral in an exercise of power in which semiotics are deployed in service of the global order [17].

Within this system the ‘qualified’ speaker is the interpreter of law, a role which is determined by professional rather than linguistic competence [17, p. 41]. Professional ‘thematic’ experts or advisers are required to ‘translate normative commitments into concrete actions’ [38], and to do so requires specific skills and normative ambition rooted in international frameworks rather than local or contextual knowledge [32]. This process of development, (re)construction and reproduction of meaning through professional practice [28, p. 51] results in a specialised language that excludes many voices and produces ignorance about them [28, p. 52]. The exclusion of non-western understandings is a symptom of underlying power structures and unipolarity which privilege the maintenance of the status quo rather than allowing for resistance or challenge [33]. This exercise of power is exemplified not only in the semiotic and pragmatic aspects of the term peace, but in the hegemonic role of the English language in the ‘translation’ of the language across diverse cultural contexts.

### Translating Peace

In principle there are six official working languages of the UN. Arabic was first recognised among them in 1973 [25]. However, over time, and in practice, English has assumed a hegemonic position. In the official forums from which normative texts emerge, debates are conducted and texts are drafted in English [25, p. 197]. This preference reflects the power dynamics within the institution and means that the starting point for ‘translation’ is not only the semantic content of the texts, but also their semiotic signifiers in English. The acceptance of technocratic approaches to peace has obscured the contestation inherent in the word, as well as related terms such as accountability, gender equality or human rights. Whereas in the professional parlance of peacebuilding the term ‘translation’ is most often used to convey the activity of implementing norms, from a linguistic perspective, the process of translation is deeply embedded in processes of meaning making. This stands in stark contrast to the technocratic approach of technical rendering of words from one language to another.

For language to be ‘translatable’ it must exist in a form that a listener/reader can understand. There is a transaction between the speaker (who owns the original meaning), the translator (as the broker), and the listener (the addressee to whom the language must be oriented). In addition to the need to be able to translate the literal meaning of words, translation must also be able to convey the intention behind the words [19, p. 228]. Translation must therefore be able to take account of socio historical knowledge that provides the context and the way in which words are understood in context. This can result in fundamental miscommunication when words or concepts simply don’t translate, or when they do not make sense outside of the situated context. ‘Translators’ – often native speakers from the country in question- are trained to render the language meaningful to the (international) addressee and become part of that epistemic community. Rarely are they afforded the opportunity to shape the broader meaning of the terms [34]. While it is often said that international activities need to be ‘localised’ or contextualised there has been little attention paid to the deeper semantic and pragmatic aspects of translation.

A central difficulty lies in the fact that English operates as the dominant sender language of UN peace vocabulary. Policy English relies heavily on compact compound expressions that condense complex political practices into technical terms. When these compounds are translated into Arabic, they encounter a linguistic system built around triliteral roots^1^ that embed social, ethical and relational meanings [26, 27]. Terms such as (ḥifẓ al-salām), preservation of peace evoke care, protection and moral duty, while (al-sulṭa al-akhlaqiyyah), moral authority draws on connotations of ethics and interpersonal obligation. Arabic translation therefore often introduces resonances that exceed the narrow bureaucratic sense intended in English policy discourse. At the same time many English compounds have no direct institutional equivalent in Arabic, creating conceptual gaps where local practices cannot be easily aligned with international categories. Translation in this environment is therefore not a neutral transfer of terminology but a political act that linguistically imposes administrative categories onto diverse lived experiences. Drawing on scholarship on Arabic morphology and semantic structuring [26, 27], the analysis presented here considers how derivational systems shape conceptual reception and how linguistic form conditions normative uptake. Translation is treated as a site of epistemic negotiation rather than mechanical equivalence. When practices grounded in local authority and social legitimacy are translated without their political genealogies, they are reduced to empty labels that cannot capture local realties including differentiated access to power or layered forms of oppression. As a result, international policy interventions such as the WPS agenda can appear linguistically detached, reinforcing the sense that English remains the authoritative source of meaning while local understandings are marginalised.

## Semantic Thickening in UN English and Its Arabic Renderings in the Women, Peace, and Security Agenda

When analysed from a linguistic perspective, the WPS resolutions construct peace through a progressively thick, juridified vocabulary of UN English. As terms such as participation, protection, prevention and gender mainstreaming accumulate across successive Security Council resolutions, they cease to operate as normative aspirations and instead acquire the force of governance mandates. What begins in UNSCR 1325 (2000) as an ethical appeal for women’s inclusion [2] is transformed by UNSCR 1820 (2008), 1889 (2009), 2122 (2013), 2242 (2015) and 2467 (2019) into a procedural architecture that assumes the existence of specialised expertise, accountability regimes, institutional restructuring, and gender-responsive financing [35, 36, 37, 38, 39]. ‘Peace’ is signified as a policy system requiring enforcement, monitoring and professional administration. The thickening of WPS terminology takes shape within an English-dominated institutional universe, where compact expressions in policy English serve as shorthand for procedural infrastructures that are presumed to exist. In this linguistic order, vocabularies of participation or protection are treated as universally meaningful even though such universality depends on legal systems, bureaucratic capacity and technical institutions that are unevenly distributed across contexts. Once translated into Arabic, this supposedly universal vocabulary must convey the normative content and implied infrastructure. It bears obligations without necessarily having corresponding institutional counterparts, producing a structural asymmetry in global gender governance. English operates as a language of authorship, while Arabic receives, adapts, and absorbs mandates whose conditions of possibility may not be locally available.

The following subsections analyse this dynamic across three axes. First, we examine how the universal crafting of women’s participation constructs a technocratic model that privileges elite, bureaucratic visibility over locally grounded authority. Second, we demonstrate how protection acquires juridified expectations in English that cannot simply be reproduced in Arabic without presupposing institutional systems that may not exist. Finally, we analyse neologisms generated through gender mainstreaming, showing how imported policy vocabulary invites backlash when it enters Arabic without historical or political genealogy.

### The Universal Crafting of Women’s Participation

Women’s ‘participation’ is one of the dominant ‘pillars’ of the WPS agenda, becoming a central signifier. Over time it has become a technocratic model of what peacebuilding should look like: a thickened policy metaphor that prescribes women’s presence in peace processes primarily through top-level appointments, advisory posts and formal decision-making positions inside UN and state bureaucratic structures. This model assumes a universal metaphor of peace and participation that risks ignoring locally grounded processes of negotiation, social legitimacy and the social genealogies through which authority is produced. This disjuncture is compounded when technical English-born compounds are translated into Arabic in ways that further accentuate perceptions of the agenda as a top-down, foreign imposition.

The universal and crafted character of WPS language can be traced in the generic lexical signifiers that have long structured UN peace vocabulary. The Agenda for Peace introduced terminologies, such as preventive diplomacy (al-diblūmāsiyyah al-wiqā’iyyah), early warning (al-indhār al-mubakkir), removal of sources of danger (izālat maṣādir al-khaṭar), peace-keeping (ḥifẓ al-salām), and (ṣunʿ al-salām) peacebuilding, that emerged alongside liberal peace frameworks as a supposedly universal language for securing peace [14]. The UN explicitly invoked a global moral authority (al-sulṭa al-akhlaqiyyah), underwriting these technical concepts as normatively binding language.

UNSCR 1325 (2000) introduces participation through formulations such as (mushārakat-hā al-kāmila) (preambular para.), literally ‘her complete participation’, referring to women’s participation within state- or UN-led structures [2]. UNSCR 1325 and 2242 extend participation into elite diplomatic spaces by recommending the appointment of women as (mumaṯṯilāt wa-mabʿūṯāt khāṣṣāt) (para. 3), ‘representatives and special envoys’ [2]. Resolution 1889 thickens this semantic field further with categories such as (taʿzīz al-dawr al-qiyādī li-l-marʾa) (para. 1), ‘strengthening the leadership role of women’, and (al-wuṣaṭāʾ al-rafīʿī al-mustawā) (preambular para.), ‘high-level mediators’, embedding women’s leadership within official bureaucratic structures [36].

Through progression over time participation becomes a governance requirement shaped by institutional expectations: state capacity, financing structures and UN authority. This accumulation of terms does not merely describe a set of practices but prescribes a normative model that privileges institutional visibility and professional credentials. This semantic complexity precedes and conditions translation. By the time the vocabulary enters other languages it is already saturated with assumptions about the lived reality of women that risk treating peace as a pre-scripted technical category rather than as a lived practice. The universal template can therefore overlook the everyday struggles of women and context-specific forms of authority and legitimacy.

Many women who exercise decisive influence in conflict settings in the Arab world (and other places) do so through social roles and localised practices that the WPS template does not capture. For example, women mediators who negotiate prisoner exchanges, secure local ceasefire corridors or coordinate community protection frequently hold substantial authority in practice, yet they rarely qualify as ‘leaders’ within formal WPS frameworks, their contributions relegated to the grass roots ‘level’ in the peacebuilding metaphor [40,41, 42]. Their authority is socially conferred through kinship networks, community trust and customary legitimacy and not conferred through top-down UN prescriptions. Yet fifty-five per cent of civil society organisations working on WPS reported that the UN prioritises partnerships with elite organisations rather than grassroots actors [43], reinforcing a model of participation that privileges UN professional standards over experiential knowledge [44]. Evidence from other domains further confirms the mismatch. In Tunisia, women farmers are central to rural livelihoods and local governance of land and livestock, yet their contributions are rarely recognised within formal peace or decision-making structures [45]. Research by ICARDA shows that Tunisian women play decisive roles in farming but are marginalised in formal leadership positions within farmer cooperatives [46].

As a result, for many women the language of UN resolutions feels remote from their daily struggles. Further, when terms travel into Arabic they arrive as highly technical compounds that do not originate within local linguistic or political traditions. This can reinforce the perception that the agenda is externally driven, top-down, and insufficiently attentive to the forms of authority and experience that shape women’s roles in conflict-affected contexts. These linguistic and institutional dynamics help to explain the backlash against WPS language observable in parts of the Arab world. The contestation is not necessarily a rejection of women’s rights as such, but a resistance to categories perceived as carrying Western genealogies that do not resonate with local histories and moral economies. Terms such as (niswiyya), feminism have acquired imported political associations, such as secularism, individual rights-based activism and Western liberal frameworks, and are therefore variably received across Arab states. Grassroots women activists in Tunisia, for example, may embrace (niswiyya) within a domestic feminist history tied to labour organising and legal reform, whereas Yemeni women mediators seldom identify with the label despite exercising significant political authority. The WPS agenda is similarly contested: pushback often targets the vocabulary and assumed universality of categories rather than the substantive aims of gender equality or women’s protection.

### Local Expectations Through Juridified Protection Obligations

The second key pillar of the WPS agenda is protection. This term functions as a liberal peace signifier that has been linguistically thickened to denote legal responsibility and institutional commitments related to sexual violence [3]. The term implies that violence will be prevented, punished and structurally addressed. As this thickening unfolds, protection within liberal peace becomes a site of political contestation, exposing the disconnect between normative discourse and the lived realities of insecurity. Once protection is framed through legal and institutional expectations, its failure is not interpreted as a structural limitation but becomes dismissible at the discursive level, where the promise of protection is treated as sufficient even when material safety is absent.

Protection as a practice is historically grounded in local realities, operating through both formal institutions and informal, customary, and community-based arrangements rooted in social legitimacy. Within the WPS agenda, however, protection develops in a way that mirrors the thickening of the peace metaphor more generally, with deep legal and security implications. In the foundational resolution, UNSCR 1325 (2000) [2], protection is framed through a humanitarian lens. Women and girls must be safeguarded from violence, displacement, and insecurity. Subsequent resolutions progressively shift protection from humanitarian care to a securitised obligation, reflecting the logic of liberal peace. In UNSCR 1820 (2008), sexual violence is explicitly recognised as a threat to international peace and security [35, para. 1], and protection becomes juridified- attached to command responsibility, military discipline, troop training, and obligations to investigate, punish, and prevent [35, para. 3]. UNSCR 1889 (2009) integrates protection into structural peacebuilding, linking safety to socio-economic rights, access to services, and institutional planning [45]. Later resolutions, 2122 (2013), 2242 (2015), 2467 (2019), embed protection within peacekeeping standards, counterterrorism responses, survivor-centred justice, and monitoring mechanisms [37, 38, 39]. By this stage, the metaphor of peace is fully thickened. Peace requires states and armed groups to exercise legal accountability, institutional oversight, and operational readiness with regards to the protection of women and girls. Protection becomes inseparable from governance, no longer a supplementary moral aspiration but a mandatory security obligation central to peace itself.

The semantic and institutional thickening of protection generates key challenges when translated into Arabic. English policy vocabulary treats ‘protection’ as shorthand for complex governance regimes, humanitarian obligations, and legal frameworks. When translated into Arabic, terms such as (ḥimāyat al-madaniyyīn) (para. 9), ‘protection of civilians’, or (al-ʿunf al-qāʾim ʿalā asās al-jins) (para. 10), ‘gender-based violence’, may convey safeguarding but cannot guarantee the institutional responsibilities implied in English. For example, OP8(c) of Resolution 1325 links protection to judicial reform, electoral systems, and policing. Its Arabic translation (ittikhādh tadābīr taḍman ḥimāyah… mā yataʿallaq minhā bi-al-dustūr wa-al-niẓām al-intikhābī wa-al-shurṭah wa-al-qaḍāʾ) (para. 8(c)) communicates the aim of safeguarding, but the institutional infrastructure that underpins such obligations in Western governance contexts is absent [2].

This tension is further visible in neologisms introduced into Arabic to replicate global protection mechanisms without engagement with local practices. Terms such as (taʿzīz al-masāʾalah) (para. 3), and (ijrāʾāt al-ʿadālah al-intiqāliyya) (para.16(d)), ‘accountability mechanisms’ related to transitional justice, and (nahj qāʾim ʿalā al-nājiyāt) (OP16), ‘survivor-centred approach’, in UNSCR 2467 presuppose specialised institutions, prosecution units, gender-responsive budgeting, and victim-centred procedures [39]. In contrast, protection in many Arab contexts is also pursued through informal arrangements. These systems operate according to relational authority, social obligations, and communal ethics rather than technical policy language. Historical forms of protection such as (al-ḥimāyah), safeguarding under one’s responsibility; (al-jiwār), protection through the granting of refuge; and (al-ʿaṣabīyah), solidarity-based collective defence, were anchored in collective responsibility (masʾūliyya jamāʿiyya), kinship, reputation (sumʿa), and moral duty (wājib). Women’s safety was mediated through gendered moral structures tied to communal honour (ʿird) and moral guardianship (wilāya), enabling collective protection while sometimes constraining autonomy [47, 48]. 2 In Yemen, Sudan, Libya, and Iraq, tribal mediation (ṣulḥ) can secure immediate safety while preventing legal prosecution, illustrating a protection logic grounded in social legitimacy rather than survivor-centred justice.

Semantic thickening therefore generates expectations that the WPS agenda has struggled to meet. Despite UNSCR 1325 obliging states to protect women and girls in conflict [2, para. 3a], implementation through National Action Plans has often been weak, under-resourced, and lacking accountability mechanisms [49]. Many states adopted NAPs but failed to budget for them, mobilise civil-society involvement, or enforce obligations [49, 50, 51 p. 301]. Moreover, the protection pillar has increasingly narrowed to sexual violence at the expense of political participation and agency, reinforcing an image of women as passive victims and sidelining transformative gendered political change [4, 52]. The thickening of protection into legal accountability and security obligations exposes the deep inequalities structuring global governance. Translation into Arabic makes visible the widening gap between the expanding semantic universe of protection in English and the limited institutional space in which this universe can materialise. The friction that emerges in translation is therefore not primarily cultural resistance. It stems from structural asymmetries in who authors global legal meaning and who is required to absorb, enact, and report on it. States most scrutinised under WPS, typically conflict-affected and aid-dependent, operate under intense international oversight and donor conditionality. They are expected to operationalise dense legal obligations without necessary institutional capacities, turning translation itself into a continuous negotiation. Broad or euphemistic Arabic formulations mitigate political risk while signalling formal compliance. In this way, the expansion of protection into legal and security mandates ultimately reveals who shapes the normative architecture of WPS and who must carry its weight. The juridification of protection under liberal peace thus creates a paradox. When global frameworks fail in practice, rights tethered to them erode. As protection becomes institutionalised through legal obligations, peacekeeping mandates, and donor conditions, women’s safety becomes rhetorically secured by infrastructures whose collapse leaves no alternative pathway for protection. When peacekeeping is withdrawn, donor priorities shift, or states lack enforcement capacity, the promise of protection collapses, leaving language intact but material security absent.

With the failure of the protection pillar of the WPS agenda comes a parallel backlash in the very semantic and normative discourses that travel with it as norms are sustained only insofar as the expectations they generate are met. Once protection is discursively framed as a legal entitlement, its non-fulfilment no longer signals the need for alternative pathways to safety but instead remains contained at the level of language, where the mere articulation of rights is treated as evidence of protection even when there are limited or non-existent material guarantees. In contexts where peacekeeping is withdrawn, donor priorities shift or states lack enforcement capacity, the promise of protection collapses, as do the discursive rights established through WPS. Thus, the erosion of liberal peace is both material and epistemic.

Regional feminist scholarship complicates any simple opposition between ‘international’ and ‘local’ vocabularies of participation. Arab feminist movements have long advanced claims to political agency, legal reform, and social transformation through genealogies grounded in anti-colonial nationalism, labour mobilisation, constitutional struggle, and religious reinterpretation rather than through the technocratic language of mainstreaming [53, 54]. In these contexts, Arab feminists frame women’s participation in everyday politics not as (only) as inclusion within existing bureaucratic structures but also as part of contestation over morality, citizenship, authority, and public legitimacy.

This literature challenges the assumption that agency must appear in recognisably liberal or institutionalised forms. As Mahmood argues, agency is not reducible to resistance to social norms but constitutes a modality of action [55, p. 157]. It may therefore be exercised through ethical self-formation, communal negotiation, or reformist reinterpretation rather than through formal policy visibility. Abu-Lughod similarly cautions against representing Muslim women as a homogeneous category requiring externally defined emancipation, highlighting the political effects of such framing [56, p. 14]. These critiques demonstrate how empowerment narratives can obscure the diverse ethical and historical registers through which women articulate political subjectivity.

Language itself constitutes a site of contestation. As Sadiqi notes, linguistic choice reflects power negotiation, with classical Arabic, colloquial registers, French, and Islamic legal vocabularies each carrying distinct political authority [57, p. 272]. Imported terminology therefore enters an already stratified semantic field and interacts with vernacular repertoires of legitimacy rather than displacing them. Recognising these epistemic traditions clarifies that resistance to WPS vocabulary does not necessarily signal rejection of women’s protection or participation. Rather, it reflects contestation over whose lexicon defines these concepts and which forms of authority are rendered legible within global peace governance. The question is not whether participation is valued, but how it is named, institutionalised, and authorised. Foregrounding these alternative traditions avoids cultural essentialism and highlights the plurality of feminist genealogies that both engage and exceed the semantic universe of UN English.

### Neologisms in Gender Governance

Gender mainstreaming is a cross-cutting theme of the WPS and one which has moved from a normative commitment to inclusion toward a dense, technical, and institutionally anchored governance framework. This institutional thickening has generated a series of complications when translated into Arabic, where many of the central concepts have no prior linguistic or political genealogy. These neologisms often appear foreign, technocratic, or externally imposed, creating conditions under which a backlash to the WPS agenda can emerge.

Gender mainstreaming, like other peace signifiers,^3^ develops across the WPS resolutions as the most technically dense and institutionally demanding dimension of the peace metaphor. In UNSCR 1325 (2000), mainstreaming appears as a normative commitment- the integration of a gender perspective into peacekeeping, humanitarian response, and post-conflict reconstruction [2]. At this early stage, gender is invoked as an ethical horizon, guiding policy but without imposing concrete bureaucratic obligations. Peace is imagined as more inclusive if gender ‘is taken into account’, yet the operational meaning of that inclusion remains vague.

This metaphor thickens considerably in subsequent resolutions. UNSCR 1889 (2009) translates gender mainstreaming into measurable institutional requirements including global indicators, systematic data collection, gender-responsive planning, and monitoring of resource allocations [36]. Gender becomes part of the administrative machinery of peacebuilding. UNSCR 2122 (2013) deepens this logic by embedding gender expertise, financing and organisational restructuring into mission mandates and UN processes [37]. By UNSCR 2242 (2015) and 2467 (2019), gender mainstreaming has become inseparable from peacekeeping and peacebuilding in various affects such as counterterrorism, security sector reform, justice reform and so on [38, 39]. The peace metaphor is now fully thickened: gender is no longer added value but a mandatory, technical, resource-intensive component of peacebuilding.

Gender mainstreaming is therefore operationalised as a bureaucratic reform mandate creating its own professional lexicon. UNSCR 1325 establishes mainstreaming as an institutional requirement, mandating gender components in peacekeeping (OP5), technical training guidelines (OP6), and system-wide reporting (OP17) [2]. UNSCR 1820 expands this to security governance, linking mainstreaming to command responsibility, disciplinary measures, vetting procedures (OP3), and justice-sector obligations such as the exclusion of sexual violence from amnesties (OP4) [35]. UNSCR 1889 (2009) embeds mainstreaming within post-conflict institution-building, requiring gender-responsive national institutions, recovery planning, sustained funding, and leadership programmes (OP8, OP10, OP11) [45]. This turn is further formalised in OP19(b–d), which requests the Secretary-General to report on mainstreaming across all planning and monitoring tools, including the development of indicators, the tracking of gender-focused allocations, and updates on the deployment of gender advisers. The Arabic formulation in OP8, (kafālat taʿmīm murāʿāt al-manẓūr al-jinsānī fī jamīʿ ʿamaliyyāt wa-qiṭāʿāt bināʾ al-salām wa-al-intʿāsh baʿd intihāʾ al-ṣirāʿ), ‘ensuring the mainstreaming of a gender perspective in all peacebuilding and post-conflict recovery processes and sectors’, reflects this institutional embedding. By Resolution 2242 (2015), these requirements extend to senior leadership accountability, system-wide conflict analysis, and gender-responsive budgeting [38].

Gender mainstreaming like other concepts emerge from liberal institutional governance, feminist bureaucracy, and rights-based frameworks. They carry epistemologies rooted in Western political imaginaries. When translating gender mainstreaming into Arabic as (taʿmīm al-manẓūr al-jinsānī), the term enters the language without its genealogies. The root (jins) previously referred to biological sex or categorisation, not to social relations. The relational suffix (ānī) was added artificially to create (jinsānī) as a translation for gender as a socio-political category. Thus, (jinsānī) is a policy neologism. Similarly, translating “gender” as (al-nawʿ al-ijtimāʿī) produces a technocratic term. These imported terms risk appearing detached from everyday linguistic and political repertoires and instead become associated with Western liberalism. This produces discursive tensions, as concepts intended as technical administrative tools may be interpreted as ideological interventions. They become vulnerable to scepticism when local experiences of violence, authority or inequality do not neatly map onto the bureaucratised frameworks implied in English.

This semantic and institutional gap has contributed to backlash in contexts where gender vocabulary enters governance through externally driven mandates rather than domestic political struggle. Feminist analyses of the WPS agenda demonstrate that its terminology is produced within institutional power hierarchies and embedded in liberal governance architectures [20, 22]. When this lexicon travels beyond the institutional site of its production, it may be reframed, diluted, or contested in order to secure local political legitimacy. Resistance to gender terminology therefore reflects not simply cultural opposition but contestation over normative authorship and epistemic authority within global governance structures [58, 12]. The backlash against neologisms such as al-nawʿ al-ijtimāʿī (‘social gender’) or taʿmīm al-manẓūr al-jinsānī (‘gender mainstreaming’) operates at the level of signification itself, where imported lexical forms are read as carriers of external political genealogies.

This resistance is not solely ideological. Disillusionment also emerges where global norms fail to produce material improvements. When WPS commitments do not translate into tangible institutional protection or participation, technocratic language becomes vulnerable to accusations of hypocrisy or neo-colonialism. The perceived foreignness of such terminology further enables political actors to challenge it on cultural or linguistic grounds, even where such objections mask efforts to preserve entrenched power structures. In this way, contestation over gender vocabulary becomes a proxy for broader struggles over sovereignty, authority, and the terms on which political legitimacy is defined.

## Conclusion

To conclude, the WPS agenda constructs peace through a semantically thick vocabulary of participation, protection, and gender mainstreaming that is inseparable from liberal peace architectures, legal enforcement, and technocratic governance. In UN English, these terms function as shorthand for dense institutional systems that presume state capacity, professionalised expertise, monitoring frameworks and sustainable financing. When this vocabulary travels into Arabic, it does not arrive as neutral language, but as a set of pre-scripted expectations on sometimes limited institutional counterparts, sitting uneasily alongside existing genealogies of authority, protection, and collective responsibility. Translation into Arabic, as presumably to other languages, therefore, does not simply transpose meaning but exposes the structural asymmetry of global governance in which normative universality is asserted from one linguistic and institutional centre, while implementation burdens and political risks are shifted elsewhere. Translation reveals the politics of WPS more clearly than the resolutions themselves. The movement of thick policy terms into Arabic does not simply transfer meaning but transforms it, compresses expectations, and at times strips or exaggerates institutional implications. Translating juridified policy compounds into a language organised around social and ethical genealogies can either overproduce moral connotations or underproduce bureaucratic precision. This process generates new semantic spaces in which local actors are required to perform compliance through borrowed terminology, while external institutions rely on textual evidence of implementation irrespective of material outcomes. At the same time, the thickening of WPS terminology does not automatically secure women’s rights. It can marginalise locally grounded practices of participation and protection, recast women’s authority as a function of elite recognition, and generate backlash against terms perceived as externally imposed or ideologically loaded. In Arabic-speaking conflict-affected contexts this produces semantic dependency, where activists and institutions must mobilise borrowed vocabularies to gain visibility and resources, even when these categories may not emerge from their own struggles. The erosion of liberal peace and responsibility-to-protect frameworks further reveals how fragile these juridified promises are: when peacekeeping mandates retreat, funding collapses or enforcement fails, the thick language of protection remains in place while material safety recedes. Attending to semantic thickening and translation is therefore not a linguistic concern alone; it is central to understanding whose experiences structure the meaning of peace, whose knowledge is rendered legible within WPS, and whose practices of peace are side-lined in the process.

If translation lays bare the asymmetries embedded within the WPS architecture, these structural dynamics also point toward possible institutional reforms. Reform cannot be confined to improved implementation alone, since the problem originates at the level of authorship and semantic authority. Multilingual drafting practices, deeper integration of linguistic and socio-legal expertise from Arabic-speaking and other contexts, and earlier negotiation of conceptual equivalence would shift norm production from unilateral projection toward shared construction. Rather than positioning Arabic as a receiving language tasked with absorbing pre-formed policy vocabulary, translation should function as a site of co-interpretation in which institutional assumptions are interrogated before they crystallise into governance mandates.

Similarly, guidance and implementation frameworks must move beyond exporting institutional templates toward sustained co-creation with nationally and locally grounded actors. Recognising socially embedded practices of participation and protection would enable WPS commitments to intersect with lived realities rather than displace them. Normative expansion must also be matched by credible commitments to capacity, financing, and enforcement, if juridified protection is not to remain discursively intact while materially fragile. Attending to translation as a constitutive dimension of global governance therefore opens a pathway toward a more reflexive and politically sustainable articulation of peace.

## Footnotes

1 For example, Arabic words often combine trilateral or quadrilateral roots with specific schemas, along with affixes (prefixes, suffixes) and clitics, contributing to very large vocabulary sizes and high out-of-vocabulary rates (Emami, & Mangu, 2008) Another study shows that Arabic morphological awareness (inflectional and derivational) develops across grades and is tied to the richness of the morphological system (Shahbari-Kassem et al. 2025).

2 Wilāya operates as moral authority in which men “protect” but also control women, mirroring how honour (ʿird) and reputation (sumʿa) frame safety through dependence. This can limit women’s autonomy or even be used to justify violence against them (Möller 2015). Yet, as Karim (2025) argues, this hierarchical model reflects a patriarchal reading, whereas the Qurʾānic concept is rooted in reciprocity rather than control.

3 For instance, in UN English, the neologism ‘peacemaking’ (United Nations 1992, paras. 15 and 40) refers to political negotiation supported by system-wide coordination, resource mobilisation, and administrative mechanisms. In classical Arabic thought, (al-silm) and (al-salām) carried ethical and spiritual connotations tied to (al-ʿadl), justice; (al-iṣlāḥ), reconciliation; and (silm maʿa Allāh), peace or harmony with God. By contrast, ‘peacemaking’ (ṣunʿ al-salām) (para. 20) and ‘post-conflict peace-building’ (bināʾ al-salām baʿd intihāʾ al-ṣirāʿ) (para. 21) are modern constructions drawn from institutional policy jargon rather than indigenous moral vocabulary [14].
